# Preliminary Studies on the Application of Grape Seed Extract in the Dyeing and Functional Modification of Cotton Fabric

**DOI:** 10.3390/biom10020220

**Published:** 2020-02-02

**Authors:** Ling Guo, Zhi-Yi Yang, Ren-Cheng Tang, Hua-Bin Yuan

**Affiliations:** 1 National Engineering Laboratory for Modern Silk, College of Textile and Clothing Engineering, Soochow University, 199 Renai Road, Suzhou 215123, China; Emily26@live.cn (L.G.); huabinyuan19@163.com (H.-B.Y.); 2 Lushan College, Guangxi University of Science and Technology, Liuzhou 545000, China; zhiyiyang666@163.com

**Keywords:** grape seed extract, tannins, cotton, antibacterial, antioxidant, UV protection

## Abstract

Cotton has the shortcomings of having no antibacterial, antioxidant and ultraviolet (UV) protection properties, which are of great importance for health protection purposes. In the present study, grape seed extract (GSE) mainly composed of proanthocyanins (tannins) was employed to simultaneously import pale colors and the three aforementioned functions to cotton fabric. The tests on the application conditions of GSE showed that pH and GSE concentration had great impact on the color depth of cotton fabric, and the color hue of dyed fabric could be controlled in the absence of pH regulators due to the weakly acidic nature of GSE solution. The fabric dyed with 10%owf (on the weight of fabric) GSE exhibited an excellent inhibition effect towards *Escherichia coli*, whereas the one dyed with 20%owf GSE had high antioxidant activity of 97%. The fabric dyed with 5%owf GSE offered excellent UV protection. This study reveals that GSE can be used as a functional finishing agent for health protection in cotton textiles in addition to coloration capability.

## 1. Introduction

Tannins are the second-largest aromatic compounds in nature and are present in various tissues of many higher plants [1]. Tannins are classified into two categories: hydrolyzable and condensed. The latter occupies a prominent position in tannin products sold globally, whereas the former accounts for about 10% [1,2]. Hydrolyzable tannins consist of gallic acid esters (gallotannins) linked to glucose or polymerized forms of hexahydroxydiphenol (ellagitannins). Condensed tannins are oligomers composed of 3–8 repetitive units of flavonoid [1].

The earliest applications are found in the field of leather and textile processing. In ancient times, tannins were used as a tanning material of leathers [3], and a colorant for the black coloring of silk, wool and cotton textiles, where the black colors result from the complexation between tannins and iron salts [4,5,6,7]. Later, more applications of tannins were further reported. Nowadays, tannins are widely used in leather tanning, wood adhesives, pharmaceuticals and medicines, additives of wine and fruit juices, flocculants of polluting materials, inhibitors of corrosion of metals, plastic resins, preservatives, and additives of flame retardant and insulating materials [3]. In recent years, the use of natural dyes as substitutes of some synthetic dyes is on the rise due to their environmental biocompatibility. Thus, tannin dyes are taken seriously again, considering their historical position in the dyeing of ancient textiles. In addition to normal coloring, the antibacterial, antioxidant, ultraviolet (UV) protection, and flame retardant properties of tannins on textiles are being utilized to develop functional textiles [7,8,9,10,11].

Grapes are one of the largest fruit crops all over the world. A high proportion (about 80%) of grapes is applied to winemaking, whereas others are consumed as raisins, table grapes, and juices [12,13]. In the process of winemaking, solid wastes accounting for about 25–30% of grapes are produced, which include grape skins and seeds [13,14]. These wastes, being the cheap source of natural phenolic compounds, contain phenolic acids (e.g., gallic acid), catechin, anthocyanins, and simple and complex flavonoids (e.g., proanthocyanidins) [15,16,17,18,19]. Due to their good bioactivities and functions [20], the extracts from the byproducts are widely used in the food, food packaging, biomedicine, and cosmetics industries [18,19]. Several researchers have reported the application of bio-colorants derived from grape pomace in the dyeing of textiles [21,22,23]. Grape pomace colorants exhibited good dyeing properties for wool fabric with accepted color-fastness, and the color depth of dyed wool depended greatly on pH and temperature [21]. Acrylic fibers could be dyed with grape pomace colorants after acrylic modification by cationization and amidoximation [22]. In another study, the extraction from fermented grape seeds, skin, and stem was employed to dye cotton fabric, and the resulting color was light reddish-brown [23]. However, the functions of these dyed textiles were not reported.

**Figure 1:**
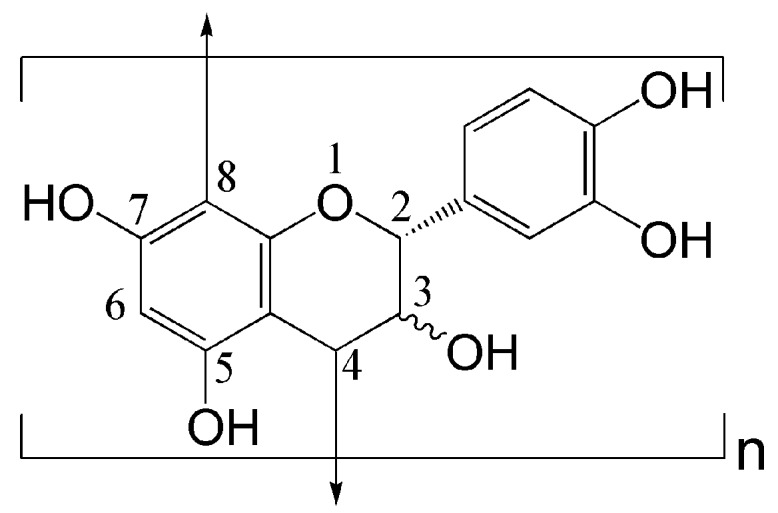
Representative chemical structure of flavan-3-ol unit and proanthocyanidins.

Proanthocyanidins (condensed tannins) are essential grape seed constituents [20], and consist of a series of polymerized flavan-3-ols which are linked principally through the 4 and the 8 positions, as shown in Figure 1 [24,25]. In consideration of previous studies on the application of tannins in textile processing, the present study aims at exploring the application of grape seed extract (GSE) containing 95% proanthocyanins in the dyeing and functional modification of cotton textile. For this purpose, the stability of the GSE solutions at different pH values and two temperatures, the dyeing conditions of GSE, and the color stability and fastness of GSE-dyed fabrics were firstly tested. Subsequently, the antibacterial, antioxidant and UV protective properties of the fabrics dyed with different concentrations of GSE were evaluated.

## 2. Materials and Methods

### 2.1. Materials

The scoured and bleached knitted cotton fabric was kindly provided by Longsheng Knitting Printing and Dyeing Co.Ltd., Jinjiang, Fujian Province, China. Grape seed extract (GSE) containing 95% proanthocyanins was purchased from Xi’an Huike Biological Co., Ltd., China. 2,2’-Azino-bis (3-ethylbenzothiazoline-6-sulphonic acid) diammonium salt (ABTS) was bought from Sigma-Aldrich (Shanghai) Trading Co. Ltd., China. Nutrient agar and nutrient broth were obtained from Sinopharm Chemical Reagent Co. Ltd., Shanghai, China, and Shanghai Sincere Biotech Co. Ltd., Shanghai, China, respectively. Citric acid, disodium hydrogen phosphate, sodium dihydrogen phosphate, potassium dihydrogen phosphate, sodium carbonate, and potassium persulfate were of analytical reagent grade.

### 2.2. Dyeing of Cotton Fabric with GSE

Cotton fabric was dipped into the GSE solution at a 50:1 bath ratio (the ratio of liquor volume to fabric weight). The dyeing was started at 20 °C, and then the solution was heated to the required temperature at a rate of 3 °C/min. At this temperature, the dyeing was continuously conducted for a set time. Afterwards, the fabric was taken out, washed thoroughly in water and air-dried.

The four dyeing conditions of GSE, including pH, temperature, time, and GSE concentration, were studied. The detailed experimental conditions are listed in Table 1. In the experiment of pH effect, the buffer consisting of citric acid and disodium hydrogen phosphate was used to adjust the pH of the GSE solution.

### 2.3. Measurements

The ultraviolet–visible (UV–vis) absorption spectra of GSE solution (0.1 g/L) at different pH values were measured using the Shimadzu UV-1800 UV–vis spectrophotometer (Shimadzu Co., Kyoto, Japan). For this study, citric acid/disodium hydrogen phosphate buffer, sodium dihydrogen phosphate/disodium hydrogen phosphate buffer, and sodium carbonate were used to adjust the pH to 4.3, 6.7, and 10.5, respectively. GSE solution was heated to 50 °C and 90 °C at a rate of 3 °C/min, and the two temperatures were kept for 60 min. Later, the GSE solution was cooled, and the spectrophotometric analysis was carried out.

The color parameters of cotton fabric were measured by the HunterLab UltraScan PRO reflectance spectrophotometer (Hunter Associates Laboratory Inc., Reston, VA, USA) using the D65 illuminant and 10 °C standard observer. Each sample was folded two times so as to get four layers, and the average of four to six measurements was reported. The following parameters were used to evaluate the colors of dyed fabric: apparent color strength (K/S), color difference (ΔE), lightness (L*), redness–greenness index (a*), yellowness–blueness index (b*), and chroma (C*) [26]. The color difference between the undyed and dyed fabrics was calculated by Equation (1) [26]:

(1) $$\Delta E=\sqrt{{\left(L_{2}^{*}-L_{1}^{*}\right)}^{2}+{\left(a_{2}^{*}-a_{1}^{*}\right)}^{2}+{\left(b_{2}^{*}-b_{1}^{*}\right)}^{2}}$$

where subscripts 2 and 1 denote the dyed and undyed fabrics, respectively.

The washing and rubbing color fastness of cotton fabrics dyed with 10% and 20%owf GSE were evaluated according to ISO 105-C06 and ISO 105-X12, respectively; for the two tests, a WashTec-P fastness tester (Roaches International, England) and a Model 670 crockmaster (James H. Heal, England) were used.

The antibacterial activity of cotton fabrics dyed without and with 5% and 10%owf GSE against *Escherichia coli* (*E. coli*) was evaluated with reference to the Chinese national standard GB/T 20944.3-2008 [27]. The fabric pieces were soaked into the conical flasks containing bacterial solutions, and then the solutions were shaken at 30 °C for 24 h. Afterwards, the bacterial solutions were diluted with sterilizing phosphoric buffer. The diluted bacterial solutions were inoculated onto the petri dishes, and cultured at 37 °C for 24 h. After bacterial culture, the bacterial colonies propagating on the petri dishes were photographed and recorded, and the antibacterial activity was calculated by Equation (2):

(2) $$\mathrm{Antibacterial}\;\mathrm{activity}\;(\%)=100\cdot \frac{N_{\mathrm{undyed}}-N_{\mathrm{dyed}}}{N_{\mathrm{undyed}}}$$

where *N*_undyed_ and *N*_dyed_ are the quantity of the visual bacterial colonies for the undyed and dyed fabrics, respectively.

The antioxidant activity of dyed cotton fabrics was evaluated by the ABTS radical decolorization method [28,29]. Firstly, the ABTS radical cation (ABTS^+^) solution was prepared by means of the reaction between ABTS (7 mM) solution and potassium persulfate (2.45 mM) and kept in the dark for about 15 h at room temperature. Prior to use, the ABTS^+^ solution was diluted with phosphate buffer (0.1 M, pH 7.4) to reach an absorbance of 0.700 ± 0.025 at 734 nm. Later, 10 mg of the fabric sample was soaked into 10 mL of the ABTS^+^ solution. After 30 min, the decolorization extent of the ABTS^+^ solution was studied by the spectrophotometric measurement. A high decolorization extent of the ABTS^+^ solution represents a high capability of the fabric to scavenge ABTS^+^, i.e., a high antioxidant activity. The decolorization rate of the ABTS^+^ solution was calculated by Equation (3):

(3) $$\mathrm{Decolorization}\;\mathrm{rate}\;\mathrm{of}\;\mathrm{ABTS}\;\mathrm{cation}\;(\%)=100\cdot \frac{A_{1}-A_{2}}{A_{1}}$$

where *A*_1_ and *A*_2_ are the absorbance of the ABTS^+^ solution before and after decolorization, respectively.

The UV protection factor (UPF) and UV transmittance of cotton fabric were determined by the Labsphere UV-1000F ultraviolet transmittance analyzer (Labsphere Inc., North Sutton, NH, USA); a single layer of the sample was tested at five different positions, and the average of the data was reported.

## 3. Results and Discussion

### 3.1. UV–Vis Absorption Spectroscopic Study of GSE

**Figure 2:**
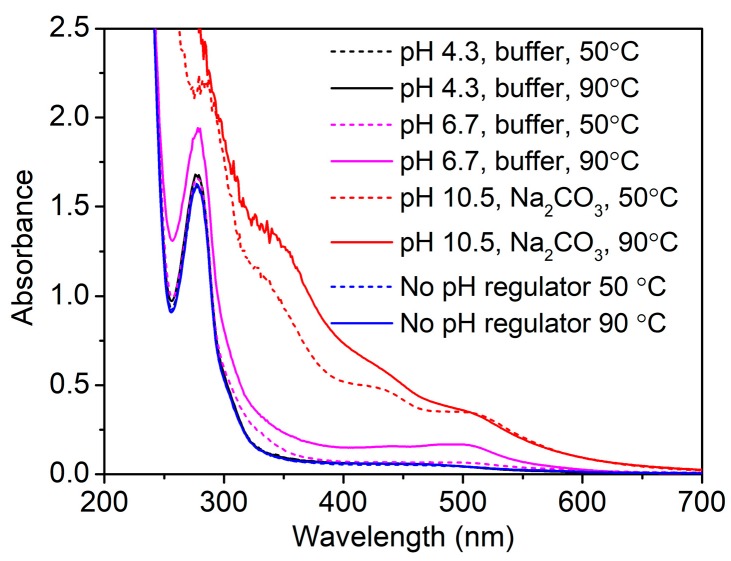
UV–vis absorption spectra of GSE solution at different pHs and two temperatures (GSE concentration, 0.1 g/L).

The main component of GSE used in this study is proanthocyanins. In an aqueous solution, phenolic hydroxyl groups in proanthocyanins easily suffer from ionization and oxidation, which can change the water solubility and stability of GSE. Thus, the spectrophotometric study of the GSE solution was carried out. Figure 2 shows the UV–vis absorption spectra of the GSE solution at different pHs, which were subjected to heat treatment at 50 and 90 °C. In the case of no addition of pH regulator, the pH of the GSE solution was 5.1 due to the ionization of phenolic hydroxyl groups in proanthocyanins. Under weakly acidic conditions (pH 4.3 (buffer) and no pH regulator), the GSE solution had a strong absorption band at 280 nm and an inflection point minimum at 258 nm. Such absorption features confirm that the main component of GSE is condensed tannins [30].

For the weakly acidic GSE solutions subjected to heat treatment at 50 and 90 °C, the differences in absorbance and spectra were very small. At pH 6.7, increasing temperature from 50 to 90 °C obviously increased the absorbance at 280 nm, and a broad absorption band at about 500 nm appeared. Furthermore, GSE solution displayed higher absorption intensity and more shoulder bands under alkaline conditions than at pH 6.7 at two temperatures. These phenomena indicate that GSE has increased solubility under neutral and alkaline conditions, and at the same time, the polyphenolic compounds of GSE are not stable at high pHs and high temperatures. The instability of GSE stems from the oxidation reaction of polyphenolic compounds at high pHs, which creates new bonds and new structures [31,32]. The spectrophotometric study suggests that the application of GSE under weakly acidic conditions exhibits good stability.

### 3.2. Application Conditions of GSE

Four application conditions of GSE (pH, temperature, time, and GSE concentration) were discussed. Because the apparent color strength (K/S) cannot reflect the color depth of dyed fabrics accurately when the changes in color occur in some cases, both K/S and color difference (ΔE) were used as evaluation indexes for studying the color of cotton fabric in the present study.

#### 3.2.1. pH

**Figure 3:**
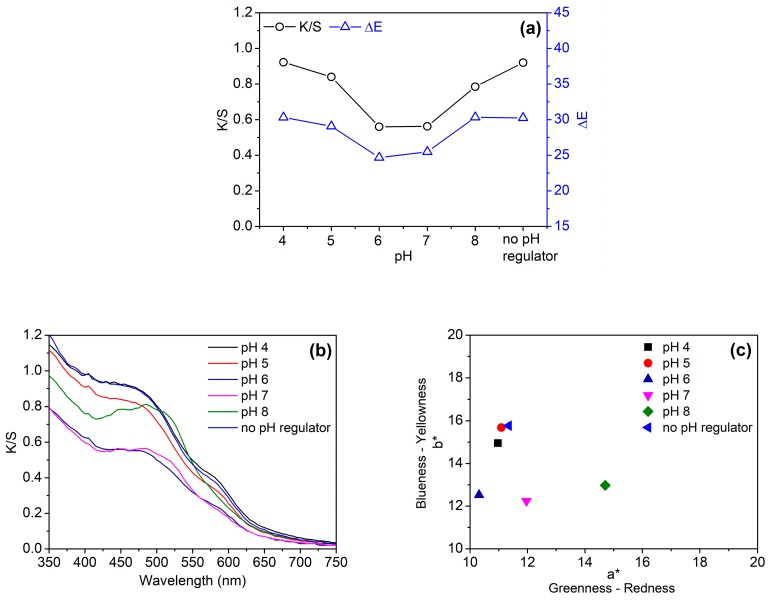
Color depth (K/S at 450 nm) and color difference (**a**), visible absorption spectra (**b**), and chromaticity coordinates (**c**) of cotton fabrics dyed with 20%owf GSE at various pH values.

Considering the effect of pH on the stability of proanthocyanins, the pH of the GSE solution was first studied. Figure 3a displays that the K/S and ΔE values of cotton fabric were low at pH 6 and 7 whereas the two values were high at pH 4 and in the absence of pH regulator. Indeed, the pH of the GSE solution used in this section was 4.5 or so due to the ionization of phenolic hydroxyl groups in proanthocyanins, although pH regulator was not added. Therefore, the color depth at pH 4 was comparable with that in the absence of a pH regulator.

The pH of the GSE solution had certain effects on the color hue of cotton fabric, as shown in Figure 3b,c. When pH was changed from 6 to 8, the maximum absorption wavelength of the dyed fabric shifted towards the long wavelength (Figure 3b). This red shift effect is caused by the ionization and oxidation of phenolic hydroxyl groups in proanthocyanins [32]. The changes in color at pH 7 and 8 were also reflected in the chromaticity coordinates of Figure 3c. The chromaticity coordinates of the fabrics dyed at these two pHs shifted more to the red color space. From Figure 3b,c, it can be observed that the visible absorption spectrum and chromaticity coordinate of the fabric dyed in the absence of pH regulator was very similar to those of the fabric dyed at pH 4. Taking the aforementioned results into consideration, a pH regulator was not used in the subsequent dyeing experiments.

#### 3.2.2. Temperature and Time

**Figure 4:**
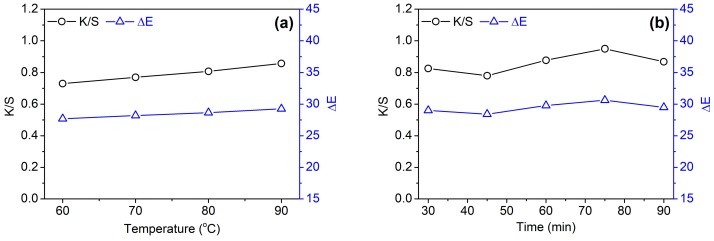
Effects of temperatures (**a**) and time (**b**) on the color depth (K/S at 450 nm) and color difference of cotton fabrics dyed with 20%owf GSE.

Figure 4a,b shows the effects of temperature and time on the K/S and ΔE values of cotton fabrics, respectively. From Figure 4a, it can be observed that the color depth of cotton fabric increased gradually when temperature was elevated from 60 to 90 °C. This phenomenon can be explained by the increased swelling of cotton fiber and the increased diffusion kinetic energy of proanthocyanins at high temperatures. Overall, the increment of color depth caused by increasing temperature is not high, which is associated with the low affinity of GSE to cotton fiber indicated by low K/S and ΔE values. This result is not completely the same as that of silk fabric dyed with condensed tannins extracted from *Dioscorea cirrhosa* tuber [7]. For the dyeing of silk, the adsorption of condensed tannins increased significantly with increasing temperature due to the high affinity of condensed tannins to silk. Similarly, time had a limited effect on the color depth of cotton fabric, although a slight increase in color depth was observed with prolonged time (Figure 4b).

#### 3.2.3. GSE Concentration

**Figure 5:**
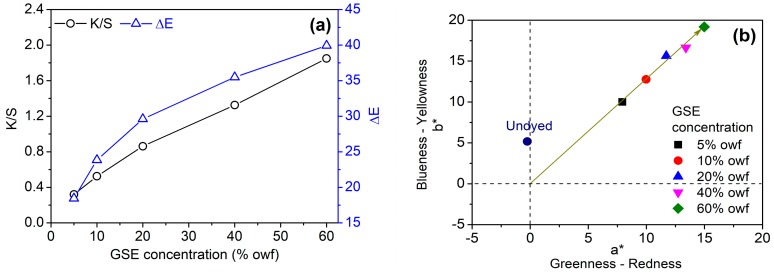
Color depth (K/S at 450 nm) and color difference (**a**), and chromaticity coordinates (**b**) of cotton fabrics dyed with GSE at various concentrations.

Figure 5a shows that the color depth of cotton fabric increased almost linearly with GSE concentration. Accordingly, the chromaticity coordinate moved upwards along a straight line (Figure 5b). This reflects the increase in chroma (C*), and also indicates the stability of color hue at different color depths.

From the above experiments, it is clear that pH and GSE concentration have a great impact on the color depth of cotton fabric. No use of a pH regulator can impart the stable color to cotton fabric, and the color depth can be adjusted by the change of GSE concentration. In such dyeing conditions, the color hue of cotton fabric is easily controlled, and the resulting colors are in the light color category.

### 3.3. Color Fastness

The color fastness of cotton fabrics dyed with 10% and 20%owf GSE was determined and are listed in Table 2. In the grade classification of fastness standards, the highest grade of washing and rubbing fastness is 5. At 10% and 20%owf GSE, the dyed fabrics reached a high level in the fastness to washing and rubbing, as expected. The good fastness is associated with the fact that the colors in the present study belong to the light category. According to the Chinese national standard GB/T 18401–2010: National General Safety Technical Code for Textile Products, the acceptable color change fastness to washing must be higher than or equal to 3–4 for baby clothing, and 3 for the textile products in direct and indirect contact with skin [33]. For the dyed fabric with 20%owf GSE, the color change fastness to washing was 3–4. This grade can meet the aforementioned requirement for color fastness.

### 3.4. Antibacterial and Antioxidant Properties

Textiles are often exposed to contamination with microbes during usage and storage. Cotton clothes are well known to have no antibacterial property. The microbial attack towards cotton textiles results in the changes in the color and appearance of fabrics, the strength loss of fabrics, unpleasant odor formation, and infectious disease. The most popular antibacterials used for textiles are metals and metal salts (e.g., silver), quaternary ammonium compounds, silane quaternary ammonium compounds, halogenated phenols (e.g., triclosan), metal-organic complexes, and polybiguanides [34]. Most of them have shortcomings of persistence in the environment, a potential for bioaccumulation, and dermal sensitization potential [34]. This is also the case for silane quaternary ammonium compounds that are extensively used for cotton products. In this regard, the eco-friendly products from plants can provide suitable alternatives [35].

**Figure 6:**
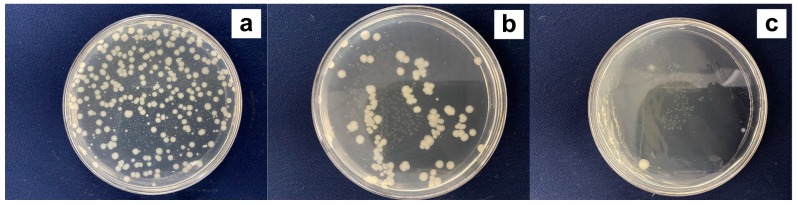
Visual bacterial cultures for undyed cotton fabric (**a**) and dyed cotton fabrics with 5% (**b**) and 10%owf (**c**) GSE.

Previous studies have demonstrated the inhibition effect of tannins from *Punica granatum*, *Quercus infectoria*, and *Dioscorea cirrhosa* towards the growth of microbes on textiles [7,8,11]. However, the antibacterial activity varied greatly with the different botanical sources and dosages of tannins [8,11]. In addition, lignins, which are similar to tannins in chemical structures, had bactericidal activity for eight bacteria cultures when applied onto linen fabric using a padding method [36]. At present, the antibacterial function of tannins from GSE for textile application remains unclear. In the present study, the inhibition effect of cotton fabrics dyed with 5% and 10%owf GSE towards the growth of *E. coli* was tested. Figure 6 shows distinct differences in the visual bacterial cultures between the undyed and dyed fabrics. For the fabric dyed with 10%owf GSE, bacterial colonies were hardly found. The sample had a high antibacterial rate of 96.3% in comparison to the undyed fabric, indicating excellent antibacterial function. In addition, the fabric dyed with 5%owf GSE displayed remarkably reduced bacterial colonies, and its antibacterial rate was 77.7%. This fabric can also be classified as an antibacterial textile according to the Chinese national standard GB/T 20944.3–2008 which requires the antibacterial products to have a bacteria inhibition rate of higher than 70% [27].

The antioxidant activity of textiles has not attracted enough attention in the past. Indeed, clothes with an antioxidant function can protect the skin against the free radicals present in the atmosphere, which are responsible for skin aging. Antioxidants can scavenge free radicals, thus deactivating the capacity of free radicals to damage the skin when incorporated into textile fibers. It is well known that the extracts from grape seeds and skins have high antioxidant capability [16,18,37]. The antioxidant property of GSE is attributed to the action of polyphenols and proanthocyanidins [37].

**Figure 7:**
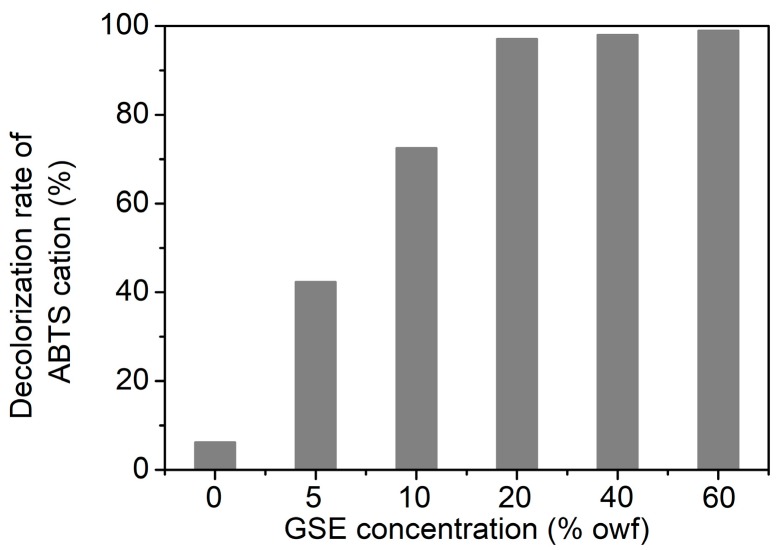
Antioxidant activity of cotton fabrics dyed with GSE at various concentrations.

Herein, the antioxidant activity of cotton fabrics dyed with GSE at various concentrations was evaluated using the ABTS^+^ decolorization assay. In this assay, the fabrics were soaked into the ABTS^+^ solution to scavenge ABTS^+^, leading to the decolorization of ABTS^+^. Thus, a high decolorization rate of ABTS^+^ represents a high antioxidant activity. Figure 7 shows that pristine cotton had no antioxidant function (decolorization rate, 6.3%). When GSE concentration increased from 5% to 20%owf, the decolorization rate of ABTS^+^ increased from 42.4% to 97.1%. At 20%owf GSE, the decolorization rate almost reached a plateau. This test proves the high efficiency of GSE on cotton fabric in scavenging free radicals.

### 3.5. UV Protection Ability

Cotton garments, which are often used in summer, cannot provide sufficient protection against solar radiation. Thus the treatment with synthesized UV absorbers containing reactive groups must be carried out [38,39]. Some reports have demonstrated that natural dyes can also impart UV protection effects to cotton textiles [40,41]. As natural compounds structurally similar to tannins, nanolignins have also been proven to be excellent UV absorbing agents for increasing the UV protection of linen fabric [36]. Such function is attributable to the strong absorption of lignins in the UV light region [42].

**Figure 8:**
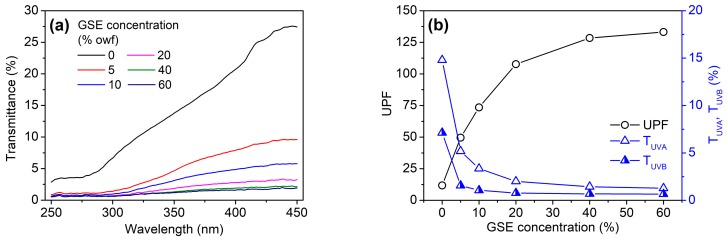
UV transmittance curves (**a**) as well as UPF and UVA/UVB transmittance (**b**) of cotton fabrics dyed with GSE at various concentrations.

Figure 8a displays the UV transmission curves of cotton fabrics. The pristine fabric showed a high transmittance in the region of both the UVB (280–315 nm) and UVA (315–400 nm), with a low UPF (11.75). The fabrics dyed with GSE showed the evident reduction in the transmittance of UVA and UVB, as indicated by Figure 8a,b. Moreover, the UV transmittance decreased and the UPF increased with increasing GSE concentration. The UPF of the fabrics dyed with 5% and 10%owf GSE was 49.56 and 73.53, respectively. Correspondingly, the UVA transmittance (T_UVA_) was 5.19% and 3.35%, respectively, whereas the UVB transmittance (T_UVB_) was 1.56% and 1.06%, respectively. In respect to the Australia/New Zealand standard AS/NZS 4399:1996 [43], the UPF rating of the two fabrics reached 40–50 and 50+, respectively, both of which are classified as “excellent protection”. The excellent UV protection capability of the GSE dyed fabric results from a number of aromatic rings present in the proanthocyanins of GSE.

## 4. Conclusions

The present study discusses the conditions of cotton dyeing with GSE as well as the color stability and color fastness of the dyed fabric and evaluates the antibacterial, antioxidant and UV protection functions of the dyed fabric. The spectrophotometric study demonstrated the good stability of GSE in a weakly acidic medium. In the case of no use of pH regulator, the color hue of dyed fabric was stable because of the weakly acidic nature of the GSE solution. Without the use of a pH regulator, the color depth of the dyed fabric was mainly dependant on GSE concentration and less affected by temperature and time. The functional properties of the dyed fabric depended on the GSE concentration. Excellent antibacterial, antioxidant, and UV protection functions were achieved at 10%, 20%, and 5%owf GSE, respectively. The present study is the preliminary exploration of the application of GSE in the simultaneous dyeing and functional modification of cotton fabric. In this study, pale colors were obtained. Further studies should be performed to increase the color depth, color fastness, and washing durability of the GSE-dyed cotton fabric.

## Figures and Tables

**Table 1:** Application conditions of grape seed extract (GSE).

Varible	Levels	Other Parameters
pH	4–8	GSE 20%owf, 90 °C, 60 min
Temperature	60–90 °C	GSE 20%owf, 60 min
Time	30–90 min	GSE 20%owf, 90 °C
GSE concentration	5–60% owf	90 °C, 60 min

Note: owf = on the weight of fabric.

**Table 2:** Color fastness of cotton fabrics dyed with GSE.

Sample	Washing			Rubbing	
	Color Change	Staining		Dry	Wet
		Cotton	Wool		
10% GSE dyed	4–5	5	5	4–5	4–5
20% GSE dyed	3–4	4–5	5	4–5	4–5

## Abbreviations

The following abbreviations are used in this manuscript:

a*	Redness-greenness index
ABTS	2,2’-Azino-bis (3-ethylbenzothiazoline-6-sulphonic acid) diammonium salt
AS/NZS	Australia/New Zealand standard
b*	Yellowness-blueness index
C*	Chroma
*E. coli*	*Escherichia coli*
ΔE	Color difference
GB/T	Chinese national standard suggested
GSE	Grape seed extract
ISO	International Organization for Standardization
K/S	Apparent color strength
L*	Lightness
owf	On the weight of fabric
UPF	Ultraviolet protection factor
UV	Ultraviolet
UVA	Ultraviolet light from 315 nm to 400 nm
UVB	Ultraviolet light from 280 nm to 315 nm
UV–Vis	Ultraviolet–visible
